# Melanoma of unknown primary in the pancreas: should it be considered primary?

**DOI:** 10.1186/s12893-020-00731-w

**Published:** 2020-04-16

**Authors:** Yanwen Jin, Congdun Ran, Fuyu Li, Nansheng Cheng

**Affiliations:** Department of Biliary Surgery, West China Hospital, Sichuan University, No. 37 Guo Xue Xiang, Chengdu, Sichuan 610041 P.R. China

**Keywords:** Melanoma of unknown primary, tumor’s origin site, Pancreatic carcinoma

## Abstract

**Background:**

Malignant melanoma is characterized as highly malignant due to its rapid growth and early metastasis. Metastatic melanoma from occult primary is rare. Melanoma of unknown primary in pancreas are even rear. But it is a biologically ill-defined and clinically understudied concept.

**Case presentation:**

In this report, a 43-year-old man was diagnosed with pancreatic carcinoma. Extended total pancreatectomy together with portal vein reconstruction and extensive lymphadenectomy were performed in our hospital. The patient was diagnosed with pancreatic malignant melanoma after pathological examination. He was still alive 20 months after the operation without any evidence of recurrence.

**Conclusion:**

The described case highlights the possibility of primary pancreatic malignant melanoma and the treatment strategies of this rare carcinoma.

## Background

Melanomas can be a challenging malignancy to diagnose and treat. They arise from the melanocytes in the cutaneous, ocular, mucosal, and unknown primaries at an incidence rate of 91.2, 5.2, 1.3, and 2.2%, respectively [1]. Melanoma of unknown primary (MUP) is characterized by the finding of metastatic melanoma within the lymph nodes, subcutaneous tissues, and other distant sites without an evident primary lesion. Recently, Song et al. reviewed the diagnosis, epidemiology, staging, etiology, treatment, outcome, and prognosis of MUP [2]. MUP is a biologically ill-defined and clinically understudied concept. Herein, we report a case of pancreatic MUP.

## Case presentation

**Fig. 1:**
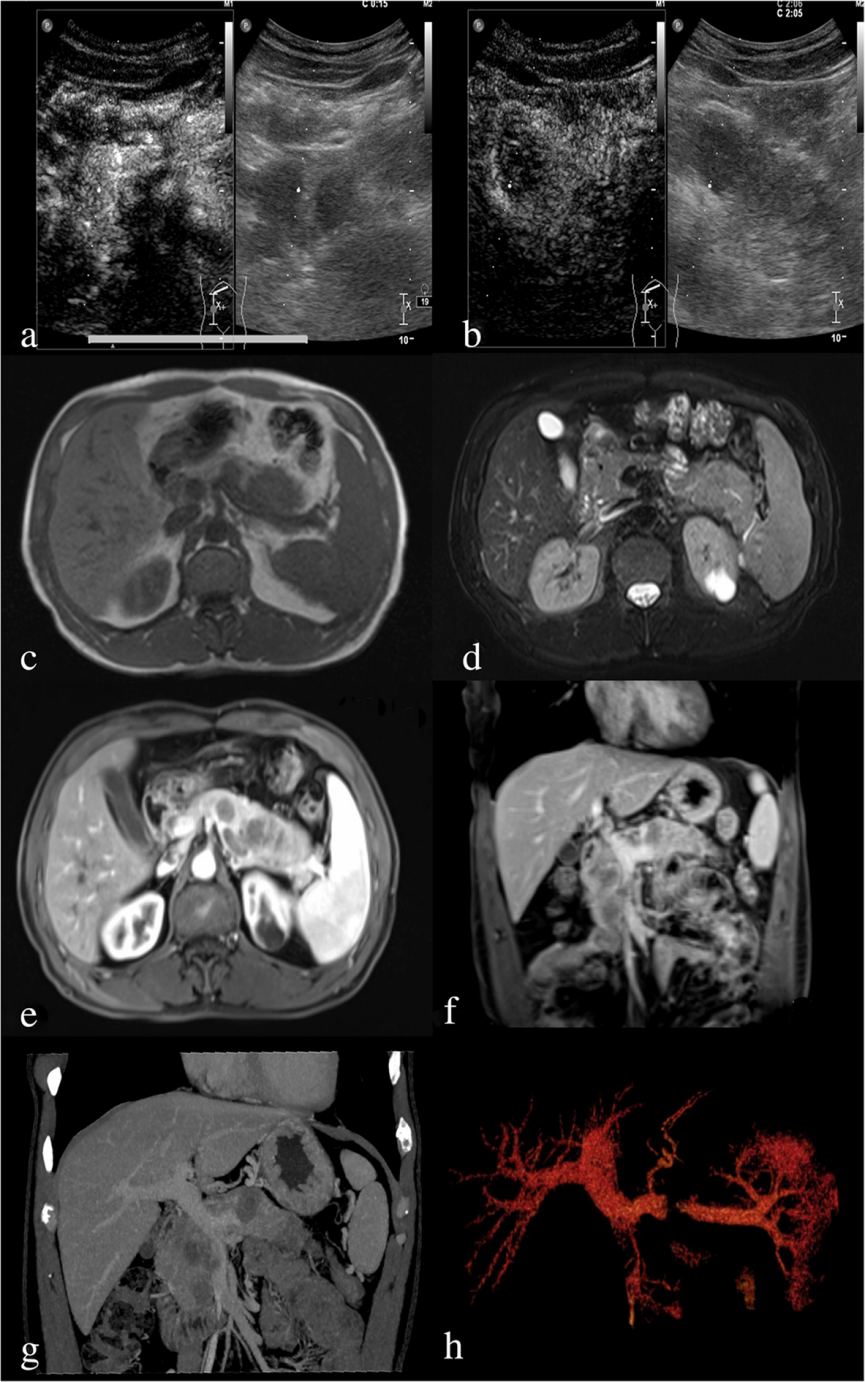
Contrast-enhanced ultrasonography (**a**, **b**), MRI (**c**, **d**, **e**, **f**) and CT (**g**) manifestation of pancreatic melanoma. Three-dimensional vascular reconstruction of portal vein system (**h**)

A 43-year-old man presented to our hospital with a 10-day history of mild epigastric pain, without weight loss or jaundice. This patient had a medical history of acute pancreatitis about 6 months before the current admission. He had a cystic neoplasm in the pancreatic tail 4 months later and was diagnosed with pancreatic pseudocysts at a local hospital. He was a non-smoker and denied any history of prior surgery. After admission, a mass was palpated on the left upper abdomen during physical examination. Routine laboratory tests results for liver function and pancreatic amylase were normal. The tumor markers of carcinoembryonic antigen, carbohydrate antigen 19–9, and carbohydrate antigen 125 were also within normal limits. Abdominal contrast-enhanced ultrasonography revealed multifocal hypoechoic mass in the pancreas (Fig. 1a and b). Magnetic resonance imaging (MRI) of the upper abdomen revealed multifocal masses occupying the entire enlarged pancreas. The lesions were hypointense on T1-weighted image (Fig. 1c) and isointense on T2-weighted image (Fig. 1d). The lesions were heterogeneously enhanced during the arterial phase and portal venous phase (Fig. 1e, f). Contrast-enhanced computed tomography (CT) also showed that these lesions were multifocal with low density and the largest one had a diameter of 2.6 cm (Fig. 1g). Three-dimensional vascular reconstruction of CT showed that a tumor thrombus formed in the superior mesenteric vein and central segment of the splenic vein (Fig. 1h).

**Fig. 2:**
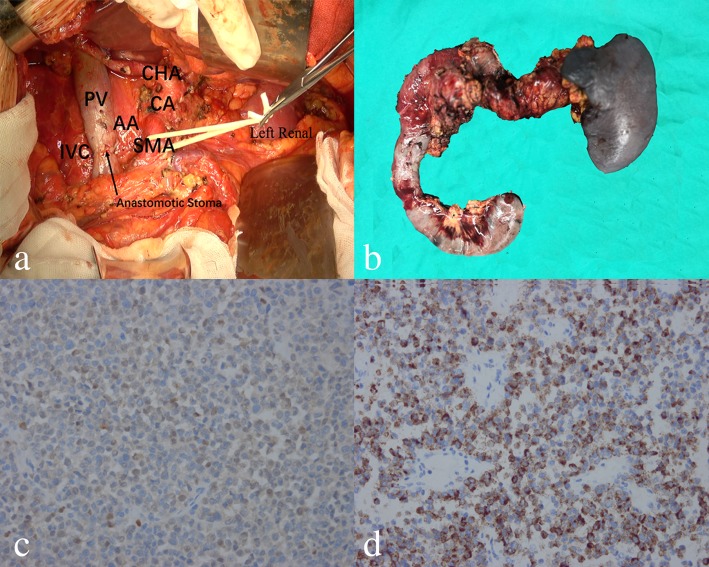
Surgical field specimen after operation (**a**, **b**). Immunostaining of the tumor cells (S-100, C; Human Melanoma Black 45, D) 400×. IVC, inferior vena cava; CA, celiac artery; PV, portal vein; AA, abdominal aorta; CHA, common hepatic artery; SMA, superior mesenteric artery

The patient was diagnosed with pancreatic carcinoma with superior mesenteric and splenic vein tumor thrombus formation. Extended total pancreatectomy together with portal vein reconstruction and extensive lymphadenectomy was performed after thorough exploration of the whole abdominal cavity (Fig. 2a, b). Pathologic examination showed surgical margins were negative. No metastasis was found within the detected lymph nodes. Immunostaining of the pancreatic tumor cells established the diagnosis of pancreatic malignant melanoma: the tumor cells were positive for Human Melanoma Black 45 (original magnification: 400×, Fig. 2c) and S-100 (original magnification: 400×, Fig. 2d) and negative for CA19–9, CA125, CK7, and CK8/18. Detailed medical history-taking and thorough physical examination including ophthalmologic, anal, genital, and nasal cavity were performed, and no other malignant location was found. Full-body positron emission tomography scan 4 months after the operation showed no evidence of malignancy. Therefore, according to diagnostic criteria set forth by Das Gupta [3], the patient was diagnosed with pancreatic MUP. The postoperative pathological stage was stage IV [4]. The patient subsequently underwent high-dose interferon-alpha 2b therapy. He was still alive 20 months after the operation without any evidence of recurrence.

## Discussion and conclusions

MUP in pancreas is rather rare. In the literature review of Nakamura [5], a total of 76 pancreatic malignant melanoma cases were reported, published in English. The major primary site was cutaneous and ocular. In 10 cases, the primary site could not be located (Table 1). No standard guidelines have been developed to differentiate between primary and secondary pancreatic malignant melanoma. Primary malignant melanoma can rarely arise de novo from migrated neural crest cells, neural cells, or melanocytes [13]. The embryonic neural crest cells can give rise to islet cell tissue. The vagal neural crest cells play an important role in differentiation of endoderm-derived organs such as the thymus, lungs, and pancreas [14]. Melanocytic differentiation and melanin pigment formation were observed in some pancreatic tumors such as solid pseudopapillary neoplasms (SPN) [15]. Accordingly, some authors have postulated a neural crest origin of SPN [16]. Theoretically, neural crest cells and melanocytes can be seen in both normal and malignant pancreases, and malignant melanoma may occur primarily in the pancreas. Coincidentally, some investigators have probed whether gastrointestinal MUP may actually represent cases of primary melanoma derived from melanoblastic cells of the neural crest [17]. In our case, the recurrence-free survival time was 20 months. We therefore speculated that the patient in our case might have primary pancreatic malignant melanoma.

**Table 1:** Overview of published cases of MUP in pancreas

Authors	Year of publication	Age	Sex	Location in the pancreas	Tumor size (cm)	Diagnostic modality	Surgery	Follow-up (month)	Outcome
Bianca et al .[6]	1991	48	M	Head	3	FNA	PD	12	Alive
Medina-Franco et al .[7]	1999	60	M	Head	8	CT, US	PD	6	Dead
Dewitt et al .[8]	2003	33	M	Head	5	EUS-FNA	Palliative gastrojejunostomy	6	Dead
		83	F	Tail	3	EUS-FNA	No operation	10	Alive
Portale et al .[9]	2011	43	F	Tail	1.7	US, CT, PET-CT	DP	NR	NR
Sperti et al .[10]	2011	48	M	Body	2.9	CT	DP	24	Dead
Goyal et al .[11]	2012	58	F	Head	10	CT-guided biopsy	PD	11.4	Dead
		69	M	Tail	4.5	Biopsy	DP	26	Dead
Ben Slama et al .[12]	2017	55	F	Head	5.5	CT, MRI	PD	15	Alive
Current	2019	43	M	Head, body, tail	-	US, CT, MRI, PET-CT	TP	20	Alive

*PD* pancreatoduodenectomy, *DP* distal pancreatectomy, *TP* total pancreatectomy, *US* ultrasonography, *EUS-FNA* endoscopic ultrasound-guided fine-needle aspiration biopsy, *NR* not report

There are few reports of clinicians’ experience with pancreatic malignant melanoma [5]. Most patients diagnosed with pancreatic malignant melanoma have undergone surgical resection. To our best knowledge, only two cases, including our own, have been treated with total pancreatectomy [18]. Curative resection of malignant pancreatic melanoma with portal and splenic vein thrombosis has not been reported in literature so far. Some researchers suggested that patients with pancreatic malignant melanoma may benefit from surgery when complete resection was possible [5, 11]. Wood et al. reported a series of 60 patients with metastatic melanoma of the gastrointestinal tract, and eight patients with metastatic lesions of the pancreas [19]. They found that six patients underwent complete surgical resection with a 5-year survival rate of 50%. Regarding the effect of lymphadenectomy on survival, some study suggested that prompt radical lymph nodes dissection led to improved survival [20]. However, the prognostic advantage of radical lymph nodes dissection could not be conclusively determined in the systematic review [21]. But lymph nodes dissection is useful for staging [4]. Patients may also benefit from adjuvant therapies including immunotherapy, chemotherapy, or radiation therapy. MUP patients appear to have equivalent or better outcomes than patients with known primaries of a similar stage [2]. In our case, radical resection combined with chemotherapy was performed in a patient with macro-vascular invasion. At the time of writing this report, the patient has survived for more than 20 months.

In conclusion, primary malignant melanoma may theoretically occur in the pancreas. More studies on patients with pancreatic malignant melanoma, as well as a standardized definition of MUP and primary pancreatic malignant melanoma are needed in future. Wide local excision and lymph node dissection combined with chemotherapy may be an optimal treatment choice for locally advanced pancreatic malignant melanoma.

## Data Availability

All data of this patient of this case report is included in this published article.

## Abbreviations

MUP: Melanoma of unknown primary
MRI: Magnetic resonance imaging
CT: Computed tomography
SPN: Solid pseudopapillary neoplasms
IVC: Inferior vena cava
CA: Celiac artery
PV: Portal vein
AA: Abdominal aorta
CHA: Common hepatic artery
SMA: Superior mesenteric artery
